# Patterns, Associated Factors, and Anatomical Concordance of Nasal and Throat *Staphylococcus aureus* Carriage Among Community-Dwelling Adults in Germany

**DOI:** 10.3390/microorganisms14051053

**Published:** 2026-05-08

**Authors:** Alexander Martens, Markus Schauer, Mohamad Motevalli, Brigitte König

**Affiliations:** 1 Institute of Medical Microbiology and Virology, University of Leipzig Medical Center, University of Leipzig, 04103 Leipzig, Germany; 2 Department of Sport Science, German University of Health & Sport (DHGS), 85737 Ismaning, Germany; markus.schauer@dhgs-hochschule.de (M.S.); moha.motevalli@dhgs-hochschule.de (M.M.)

**Keywords:** community carriage, colonization, staphylococcal ecology, pathogen reservoirs, infection, antimicrobial resistance, multidrug-resistant organisms, resistance surveillance, molecular epidemiology, microbiology

## Abstract

Despite its clinical importance, *Staphylococcus aureus* colonization in community populations remains insufficiently understood. This study aimed to determine the prevalence, anatomical distribution (nasal versus throat), and antimicrobial resistance patterns of *Staphylococcus aureus* colonization in healthy community-dwelling adults and to identify demographic and clinical factors associated with carriage. A cross-sectional study was conducted among 100 community-dwelling adults in Germany, yielding 200 nasal/throat samples. Staphylococcal isolates were identified using MALDI-TOF MS, and antimicrobial susceptibility was determined according to EUCAST guidelines. MRSA and PVL genes were assessed using molecular assays, and genetic relatedness was evaluated by rep-PCR. Associations with demographic and clinical variables were analyzed using multivariable logistic regression in R. *Staphylococcus aureus* carriage prevalence was 39%, higher in the nose (33%) than the throat (19%), with rare MRSA (3%) and no PVL detection. Significant nasal–throat discordance was observed (*p* < 0.01), with a fair agreement between sites (κ = 0.34). Resistance patterns among *Staphylococcus aureus* isolates were dominated by penicillin G resistance (47%), while 35% remained fully susceptible, and multidrug resistance was rare (6%). Multivariable analyses indicated no strong associations between overall, nasal, or throat carriage and age, sex, recent antibiotic use, or other clinical exposures (*p* > 0.05), with wide confidence intervals, potentially reflecting limited statistical power and only modest model discrimination (AUC 0.65–0.68). These findings indicate that community *Staphylococcus aureus* colonization is potentially marked by modest prevalence, substantial anatomical discordance, and a low-risk resistance profile, while common demographic and clinical factors contributed little to explaining carriage patterns.

## 1. Introduction

The World Health Organization (WHO) has repeatedly emphasized the accelerating threat posed by resistant pathogens and the urgent need to understand reservoirs of colonization within the general population [1]. Among these pathogens, *Staphylococcus aureus* (*S. aureus*) occupies a central position due to its dual role as a common commensal organism and a major cause of community-acquired and healthcare-associated infections [2]. However, key aspects of its colonization ecology (particularly the distribution of anatomical reservoirs in healthy individuals and the contribution of non-nasal sites) remain insufficiently characterized, despite their relevance for transmission and infection risk.

*S. aureus* was first described in the late 19th century, and its taxonomic distinction from coagulase-negative staphylococci (CNS) laid the foundation for modern clinical microbiology [2,3,4]. These Gram-positive, catalase-positive cocci exhibit remarkable environmental resilience, including tolerance to desiccation and high salt concentrations [5,6,7]. As a commensal, *S. aureus* colonizes multiple anatomical sites, particularly the anterior nares, throat, and skin [8]. Approximately 20–30% of adults are persistent carriers, while in 60–70% colonization occurs intermittently [9,10,11,12]. Although colonization is typically asymptomatic, it represents a critical precursor to infection, with *S. aureus* responsible for a wide spectrum of clinical manifestations, including skin and soft-tissue infections, pneumonia, bacteremia, and device-associated infections [2,13].

Epidemiologically, *S. aureus* carriage is widespread in the general population, with the nasal cavity traditionally regarded as the primary reservoir [14]. However, growing evidence indicates that the throat is an equally important, and in some individuals, it is the dominant site of colonization [15,16,17]. Evidence shows that up to one-third of carriers may be positive exclusively in the throat, meaning that nasal sampling alone can substantially underestimate true prevalence [18,19,20]. Combined nasal and throat sampling therefore provides a more accurate assessment of colonization patterns and enhances detection sensitivity, particularly in community settings where colonization dynamics may differ from clinical environments.

Coagulase-negative staphylococci (CNS) also play an increasingly recognized role in the ecology of staphylococcal colonization and antimicrobial resistance [21,22]. Although historically regarded as less pathogenic, CNS are now major contributors to device-associated infections and bloodstream infections, and many species harbor mobile genetic elements (such as SCCmec), which encode resistance determinants, including *mecA* [23,24,25]. Their ubiquity on skin and mucosal surfaces makes them important reservoirs for resistance genes that may be transferred to *S. aureus* or other pathogens [26,27]. However, antimicrobial resistance patterns of *S. aureus* in community-dwelling adults remain inadequately described, particularly outside hospital-based settings where most available data originate.

In addition to phenotypic resistance profiling, molecular typing methods such as repetitive-element PCR (rep-PCR) offer insights into the genetic relatedness of colonizing strains [28,29]. Rep-PCR generates strain-specific banding patterns based on repetitive chromosomal elements and has been standardized for inter-laboratory comparison [29,30]. Although not intended for phylogenetic reconstruction, rep-PCR provides a practical screening-level tool for assessing clonal similarity within community populations [31]. Such analyses may help identify whether colonizing strains circulate within specific community environments or reflect independent acquisition events.

The emergence and dissemination of methicillin-resistant *S. aureus* (MRSA) further highlight the importance of understanding colonization in healthy populations [32,33]. MRSA strains carry the *mecA* gene encoding PBP2a, conferring resistance to nearly all β-lactam antibiotics [34,35]. Although MRSA prevalence in German hospitals has declined in recent years [36], community reservoirs remain relevant for transmission and potential infection. Colonizing strains in healthy adults may serve as precursors to invasive disease or act as vehicles for the spread of resistance determinants. Hence, characterizing antibiotic susceptibility patterns, including multidrug-resistant (MDR) phenotypes, among colonizing *S. aureus* isolates in healthy adults is therefore essential for public-health surveillance.

Transmission of staphylococci is facilitated by close interpersonal contact, shared surfaces, and inadequate hygiene practices [37]. Environments such as sports clubs and rehabilitation facilities provide opportunities for microbial exchange across diverse age groups and social backgrounds [38,39]. In particular, fitness studios represent high-contact environments where shared equipment and frequent skin-to-surface interactions may promote colonization and exchange of staphylococcal species [40,41]. While demographic and clinical factors (including age, sex, and recent antibiotic exposure) may additionally influence colonization risk [42,43], colonization patterns in physically active, community-dwelling adults remain understudied.

Despite extensive research on *S. aureus* colonization, several gaps remain understudied. Most epidemiological studies have been conducted using nasal sampling alone or have focused on clinical populations [15,42,44,45,46], whereas combined nasal and throat colonization patterns among healthy adults have been comparatively underexplored. Data on species-level CNS colonization, antibiotic resistance profiles, and the influence of demographic factors such as sex and age are likewise limited [45,46,47]. Overall, current evidence is fragmented and does not provide a comprehensive, site-specific understanding of staphylococcal colonization in healthy community populations. The present study aimed to characterize the epidemiological, microbiological, and molecular features of nasal and throat colonization by *S. aureus* and CNS in healthy community adults. Specifically, (i) the prevalence and species distribution of colonization at each anatomical site were quantified; (ii) concordance and discordance between nasal and throat carriage were evaluated; (iii) demographic (including age and sex) and clinical predictors were examined; and (iv) antibiotic susceptibility profiles, MDR phenotypes, and the genetic indicators of *S. aureus* isolates were assessed.

## 2. Materials and Methods

### 2.1. Study Design and Participants

The present cross-sectional study was designed to investigate nasal and throat colonization with *S. aureus* and CNS among healthy adults. The study was conducted in accordance with the Declaration of Helsinki and applicable national regulations. No additional ethical approval was required for the retrospective use of the data. Participant anonymity was ensured by assigning unique identification codes and storing personal identifiers separately from microbiological and analytical data. Written informed consent had been obtained from all participants prior to data collection.

Participants were recruited from a fitness studio in Munich, Germany. Those who were regular visitors to the fitness studio and able to perform self-sampling according to standardized instructions were eligible for inclusion. Exclusion criteria comprised acute respiratory infection at the time of sampling, current hospitalization, or inability to provide informed consent. Sample collection took place over the period from October to December 2017. A total of 100 adults were enrolled, each providing one nasal and one throat swab, resulting in 200 human specimens for microbiological analysis. The sample size corresponds to a pragmatic sampling strategy commonly used in exploratory colonization studies.

### 2.2. Data Collection

Participants completed a structured questionnaire, collecting data on demographic characteristics (age, sex) and epidemiological variables associated with staphylococcal colonization, such as the presence of chronic medical conditions, recent antibiotic therapy, recent hospital treatment, skin diseases, and occupational exposure to healthcare or agriculture. Variables were selected based on established risk factors reported in the literature and were used to explore associations with overall, nasal, and throat *S. aureus* carriage.

Nasal and throat samples were collected in line with standardized instructions [48]. Sterile Copan™ E-Swab™ systems were used for both anatomical sites, and swabs were moistened with transport medium prior to sampling. For nasal specimens, the swab was inserted sequentially into both anterior nares and rotated gently along the mucosal surface. Throat specimens were obtained by swabbing the posterior pharyngeal wall while avoiding contact with the tongue and buccal mucosa. All samples were transported to the microbiology laboratory under appropriate conditions and processed within the recommended time frame.

### 2.3. Microbiological Culture and Isolation

From each E-swab, 10 µL of transport medium was inoculated onto Müller–Hinton agar with 5% sheep blood and mannitol salt agar selective for staphylococci. Plates were incubated at 37 °C for approximately 24 h. Colonies with morphology consistent with staphylococci were subcultured onto blood agar to obtain pure isolates. Presumptive *S. aureus* isolates were initially screened using a latex agglutination coagulase test. Species-level confirmation of *S. aureus* and all other staphylococcal isolates, including coagulase-negative staphylococci (such as *S. epidermidis*, *S. hominis*, and *S. haemolyticus*), was performed using matrix-assisted laser desorption and ionization time-of-flight mass spectrometry (MALDI-TOF MS) by comparing protein mass spectra with validated reference databases. Confirmed isolates were stored at −80 °C in cryobank tubes for subsequent analyses.

### 2.4. Methicillin-Resistant S. aureus

Methicillin resistance among *S. aureus* isolates was assessed using both phenotypic and molecular methods. Initial screening was performed on MRSA chromogenic agar. Phenotypic determination of methicillin resistance was conducted using oxacillin disk diffusion testing. Molecular confirmation of MRSA was conducted using the Xpert MRSA G3 assay (Cepheid, Sunnyvale, CA, USA), targeting the *mecA* gene complex. In addition, the Panton–Valentine leukocidin (PVL) gene [49] was analyzed to identify potential community-associated MRSA strains.

### 2.5. Antibiotic Susceptibility Testing

Antibiotic susceptibility testing was performed for all *S. aureus* isolates using the disk diffusion method on Müller–Hinton agar, in accordance with EUCAST clinical breakpoints applicable to the study period [50]. The antimicrobial panel comprised more than 20 agents relevant to the treatment of staphylococcal infections, including representatives of β-lactams, macrolides, lincosamides, fluoroquinolones, tetracyclines, aminoglycosides, glycopeptides, and trimethoprim–sulfamethoxazole. *S. aureus* ATCC 25923 was used as a quality-control strain. Zone diameters were interpreted according to EUCAST criteria [50], and isolates were categorized as susceptible, intermediate, or resistant. Resistance profiles were summarized for individual agents and, where appropriate, for MDR patterns. MDR was defined as acquired non-susceptibility to at least one agent in three or more antimicrobial classes [51]. Antibiotic susceptibility outcomes for *S. aureus* were documented at the level of both single-agent resistance (e.g., penicillin G, tetracycline, erythromycin) and composite resistance profiles spanning multiple antibiotic classes (e.g., cefotaxime-based or fusidic acid-associated resistance patterns), enabling characterization of isolated resistance as well as infrequent MDR phenotypes.

### 2.6. Repetitive-Element PCR (Rep-PCR)

Rep-PCR was employed as a screening-level method to assess genetic similarity among selected *S. aureus* isolates from human and, in the broader project, environmental samples [52,53]. Genomic DNA was extracted from pure cultures using standard procedures according to the manufacturer’s instructions. Amplification targeted repetitive extragenic palindromic (REP) sequences. PCR products were separated by agarose gel electrophoresis and visualized under ultraviolet illumination after staining with a nucleic acid dye. Digital images of the gels were analyzed using band-pattern comparison software, and dendrograms were generated based on similarity coefficients (e.g., Dice coefficient) and the unweighted pair group method with arithmetic mean (UPGMA). Rep-PCR was interpreted as a low-resolution typing method suitable for identifying potential clustering patterns but not for high-resolution phylogenetic inference or definitive transmission mapping. Genetic relatedness among *S. aureus* isolates was inferred from rep-PCR banding patterns, with clusters defined at a ≥85% similarity threshold.

### 2.7. Statistical Analysis

All statistical analyses were conducted in R (version 4.5.1) using established packages for data management, descriptive analysis, comparative statistics, regression modeling, and data visualization. Participant characteristics and *S. aureus* carriage outcomes were summarized using medians and interquartile ranges for continuous variables and counts with percentages for categorical variables. Group differences in categorical variables were assessed using Fisher’s exact test, and age distributions were compared using the Wilcoxon rank-sum test. Anatomical concordance between nasal and throat carriage was evaluated by cross-tabulation, McNemar’s test (including the exact McNemar test), and agreement statistics, namely Cohen’s kappa and prevalence- and bias-adjusted kappa (PABAK), each with 95% confidence intervals (95% CI). Co-occurrence patterns of nasal and throat carriage were visualized using a heat-mapped contingency table and a Sankey diagram. Determinants of *S. aureus* carriage were examined using three multivariable logistic regression models for overall, nasal, and throat carriage. Candidate predictors included age, sex, and recent antibiotic therapy; additional clinical variables (blood pressure medication, inpatient treatment, healthcare-worker status, agricultural contact, and acute wounds) were incorporated in the full model used for the forest plot. Adjusted odds ratios (ORs) with 95% CI and *p*-values are reported. Effect modification by sex was assessed by including an age-by-sex interaction term and comparing models with and without the interaction using likelihood ratio tests. Model fit (AIC) and discrimination (AUC) were used to summarize model performance. Results with *p*-values below 0.05 were interpreted as statistically significant.

## 3. Results

The present study included 100 participants (65 females and 35 males); the median age was 34 years (Q1–Q3: 26–55) with no difference by sex (*p* = 0.9). Age group distribution showed a borderline sex difference (*p* = 0.050), with males more often in the 35–49-year category (29% vs. 9.2%) and females more frequently ≥65 years (23% vs. 8.6%). Inpatient treatment tended to be more common among females (25% vs. 8.6%; *p* = 0.051), whereas other comorbidities and exposures, including diabetes (3.1% vs. 2.9%; *p* > 0.9), blood pressure medication (15% vs. 11%; *p* = 0.8), recent antibiotic therapy (14% vs. 17%; *p* = 0.7), and healthcare-worker status (28% vs. 26%; *p* = 0.8), did not significantly differ by sex (Table 1).

The overall *S. aureus* carriage was observed in 39% of participants (24/65 females, 37%; 15/35 males, 43%; *p* = 0.6). Nasal carriage was detected in 33% (29% of females vs. 40% of males; *p* = 0.3), while throat carriage was present in 19% (23% vs. 11%; *p* = 0.2). MRSA positivity was rare (3.0%), occurring only among females (4.6% vs. 0%; *p* = 0.5). All MRSA isolates were recovered from nasal swabs. PVL genes were not detected, indicating healthcare-associated MRSA profiles. Regarding *S. aureus* carriage patterns, 61% were non-carriers (63% of females vs. 57% of males), 20% had nasal-only carriage (14% vs. 31%), 6.0% throat-only carriage (7.7% vs. 2.9%), and 13% concurrent nasal and throat carriage (15% vs. 8.6%), with no statistically significant difference overall (*p* = 0.2) (Table 1).

Across the paired nasal–throat samples, 61 participants tested negative on both sites, and 13 tested positive on both sites. Discordant results were observed in 20 nasal-positive/throat-negative pairs and 6 nasal-negative/throat-positive pairs. McNemar’s test indicated a statistically significant difference between the discordant categories (*p* = 0.0108), which was consistent with the exact McNemar test (*p* = 0.006). Overall agreement between sampling sites was fair (kappa = 0.34), consistent with standard interpretation thresholds [54], where κ values between 0.21 and 0.40 indicated fair agreement, and the agreement improved to moderate after adjusting for prevalence and bias (PABAK = 0.48; standard threshold: 0.41–0.60).

**Figure 1:**
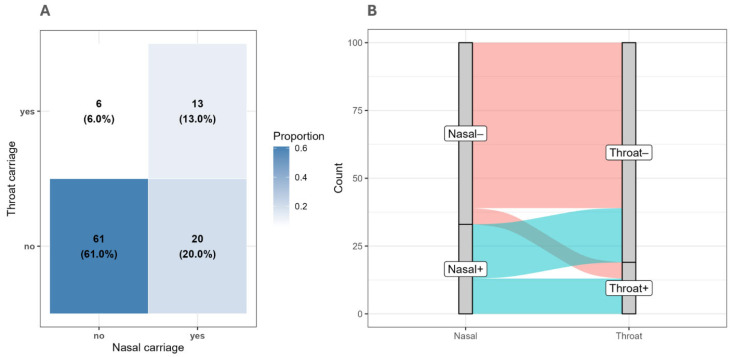
Distribution of nasal and throat carriage of *S. aureus*: (**A**) Heat-mapped contingency table showing the joint distribution of nasal and throat carriage status, with cell shading proportional to the relative frequency of each combination. (**B**) Sankey diagram illustrating the flow of participants between nasal and throat carriage categories, with flow width representing the proportion of individuals in each pathway.

To summarize the co-occurrence patterns of nasal and throat carriage, two complementary descriptive visualizations were generated. A heat-mapped contingency table was constructed to display the joint distribution of carriage status across both anatomical sites, with cell shading scaled to the relative frequency of each category combination. A Sankey diagram was also produced to illustrate the flow between nasal and throat carriage categories, with flow width representing the relative frequency of each pathway. Figure 1 shows a descriptive overview of nasal and throat carriage patterns, combining a joint distribution display with a visual representation of flow between the two sites.

**Figure 2:**
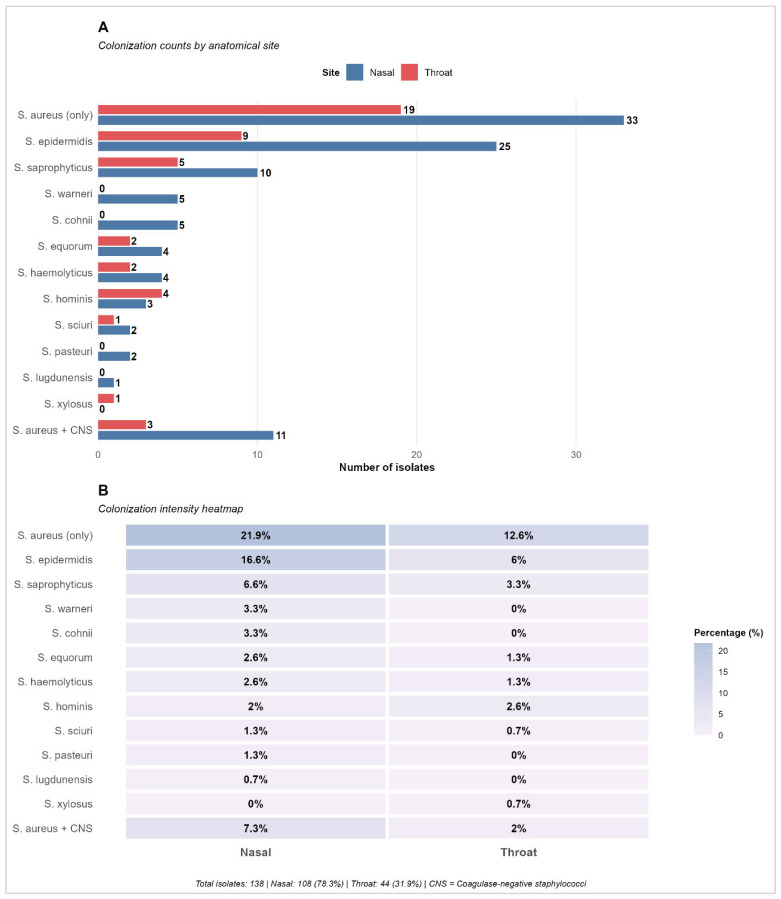
Colonization patterns of Staphylococcus species: (**A**) Colonization counts by anatomical site. (**B**) Colonization intensity heatmap showing percentage distribution.

Extending the analysis beyond *S. aureus*, the overall distribution of Staphylococcus species was also assessed. Figure 2 shows that nasal samples contributed the majority of isolates (108/138, 78.3%), compared with 44 (31.9%) from the throat. *S. aureus* (only) was the most frequent species at both sites, with 33 nasal and 19 throat isolates, corresponding to the highest colonization intensities (21.9% and 12.6%, respectively). *S. epidermidis* was the second most common species, again predominating in the nose (25 nasal vs. 9 throat isolates; 16.6% vs. 6.0%). A range of other coagulase-negative staphylococci (e.g., *S. saprophyticus*, *S. warneri*, *S. haemolyticus*, and *S. hominis*) were detected at lower frequencies, with consistently higher percentages in nasal than throat samples. Co-colonization with *S. aureus* plus CNS was observed but remained relatively infrequent compared with single-species carriage.

Antibiotic resistance patterns in *S. aureus* were further characterized. Antibiotic susceptibility testing of all *S. aureus* isolates showed that 11 isolates (35%) were fully susceptible to all tested agents. Among resistant isolates, single-class resistance predominated, most frequently to penicillin G (47%), followed by a single isolate resistant to tetracycline (3%). Resistance involving two antimicrobial classes was uncommon and observed in three isolates (9%). MDR was rare and identified in two isolates (6%). These included one isolate exhibiting resistance to penicillin G, erythromycin, and fusidic acid and another displaying a broader resistance profile involving cefotaxime, tetracycline, penicillin G, erythromycin, and fusidic acid. Stratification by anatomical site showed a comparable distribution of fully susceptible isolates between nasal (n = 5) and throat (n = 6) samples. Penicillin resistance was more frequently observed among nasal isolates (10 vs. 5) (Table 2).

**Figure 3:**
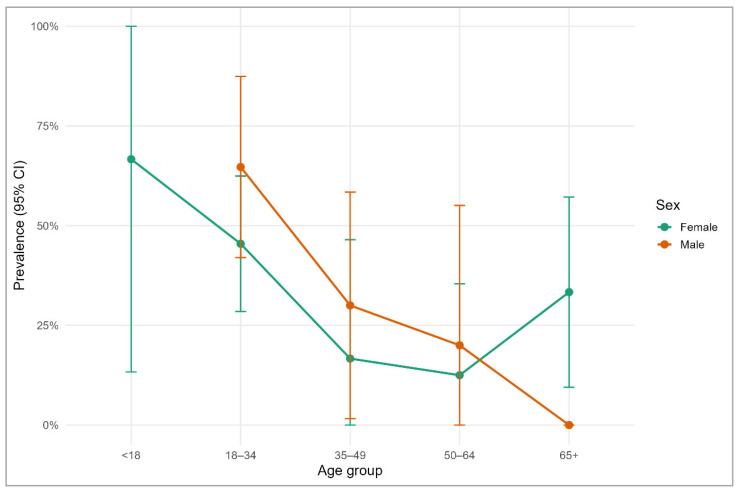
Prevalence of *S. aureus* carriage by age group and sex, with 95% CI. Prevalence estimates were calculated separately for males and females across five predefined age categories.

Prevalence of *S. aureus* carriage varied across age groups and showed modest differences between males and females. The highest prevalence was observed among participants aged 18–34 years, with both sexes showing a similar pattern of elevated carriage in early adulthood. Prevalence declined in the 35–49 and 50–64 age groups, although confidence intervals overlapped substantially. Among participants aged 65 years and older, females showed a slight increase in prevalence, whereas males demonstrated a marked decrease, though estimates were imprecise due to smaller sample sizes (Figure 3).

**Figure 4:**
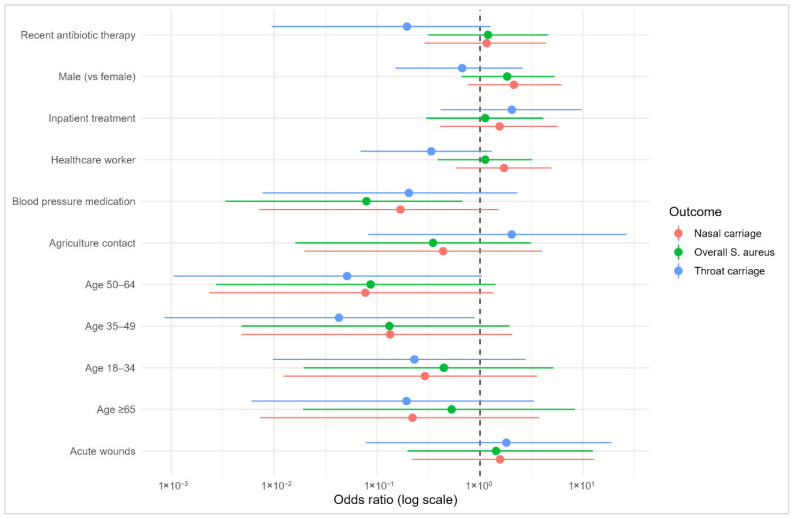
Forest plot of adjusted odds ratios (ORs) and 95% confidence intervals from the full multivariable model assessing predictors of overall, nasal, and throat *S. aureus* carriage. The model includes age category, sex, recent antibiotic therapy, blood pressure medication, inpatient treatment, healthcare-worker status, agriculture contact, and acute wounds. ORs are displayed on a logarithmic scale.

In the full multivariable model (Figure 4), no strong associations were observed between the examined predictors and *S. aureus* carriage across anatomical sites. When age was modeled categorically, prevalence patterns showed no consistent gradient for overall, nasal, or throat carriage, and confidence intervals were wide across all age groups. Sex, recent antibiotic therapy, blood pressure medication, inpatient treatment, healthcare-worker status, agricultural contact, and acute wounds similarly demonstrated imprecise estimates with confidence intervals spanning the null.

To further explore potential demographic influences, a focused model examining key determinants (including age, sex, recent antibiotic therapy, and the age–sex interaction) was fitted and is presented in Table 3. Age showed odds ratios close to unity for overall (OR 0.98, 95% CI 0.96–1.01; *p* = 0.20), nasal (OR 0.98, 95% CI 0.95–1.01; *p* = 0.20), and throat carriage (OR 0.98, 95% CI 0.95–1.01; *p* = 0.30), indicating no clear directional effect. Males exhibited higher point estimates than females for overall (OR 9.11, 95% CI 0.75–163; *p* = 0.10), nasal (OR 6.46, 95% CI 0.55–103; *p* = 0.20), and throat carriage (OR 3.21, 95% CI 0.08–435; *p* = 0.60), although the wide confidence intervals reflect substantial imprecision. Recent antibiotic therapy showed no meaningful association with overall (OR 1.30, 95% CI 0.39–4.20; *p* = 0.70) or nasal carriage (OR 1.30, 95% CI 0.37–4.25; *p* = 0.70), while a lower odds of throat carriage was suggested (OR 0.25, 95% CI 0.01–1.47; *p* = 0.20) but remained statistically inconclusive. The age–sex interaction term did not indicate a meaningful effect modification for overall (OR 0.95, 95% CI 0.88–1.01; *p* = 0.12), nasal (OR 0.96, 95% CI 0.89–1.02; *p* = 0.30), or throat carriage (OR 0.94, 95% CI 0.80–1.04; *p* = 0.40). Model discrimination was modest (AUC 0.65–0.68), reflecting the limited predictive performance of the included demographic and clinical determinants in distinguishing carriage status.

To characterize the genetic relatedness of the *S. aureus* isolates, rep-PCR typing was performed on 52 strains (Supplementary Materials: Figures S1 and S2). Rep-PCR analysis identified four major clusters with ≥85% similarity. Cluster 3 was the largest, comprising 31 isolates that were further subdivided into three subclusters, indicating some within-cluster heterogeneity. As rep-PCR reflects genetic similarity rather than formal phylogenetic relationships, these patterns are consistent with moderate strain diversity within the study population. No cluster showed a clear association with specific demographic characteristics or colonization site.

## 4. Discussion

In the present cross-sectional study of community-dwelling adults, *S. aureus* carriage at the anterior nares and throat was quantified, CNS were characterized, antibiotic susceptibility was profiled, demographic determinants were evaluated, and the genetic relatedness of *S. aureus* isolates was assessed using rep-PCR. The main findings were as follows: (i) an overall *S. aureus* carriage prevalence of 39%, with higher detection in the nose (33%) than the throat (19%) and rare MRSA carriage (3%), all PVL-negative and nasal; (ii) marked nasal–throat discordance in paired sampling, alongside *S. aureus* isolates showing largely favorable susceptibility patterns with penicillin G resistance predominance (47%) and infrequent MDR (6%); and (iii) no significant associations with age, sex, or recent antibiotic use in multivariable models, with rep-PCR indicating moderate strain diversity without clustering by site or demographic factors.

The present study identified an overall *S. aureus* carriage prevalence of 39%, confirming the anterior nares as the dominant colonization site (33%). These findings are broadly consistent with large-scale epidemiological estimates, which report carriage in approximately 20–30% of individuals [9,10,11,12,55]. The slightly elevated prevalence observed here may reflect the specific exposure environment (fitness facility), where repeated skin-surface contact and interpersonal proximity may facilitate transmission, as suggested by previous environmental microbiome studies [40,41]. From a mechanistic standpoint, nasal predominance is likely driven by niche-specific adhesion factors and immune evasion strategies, whereas throat colonization may depend more strongly on microbiome interactions and mucosal immunity [8,17]. The 3% MRSA prevalence aligns with the generally low community MRSA carriage (2.6%) reported in a large-scale study [55]. While the prevalence of MRSA may vary across settings and sampling frames, the observed PVL-negative profiles may reflect healthcare-associated lineages rather than classic community-associated MRSA (CA-MRSA) [9,27,56]. Collectively, surveillance and screening strategies in community settings should not assume equivalence between sites; relying on nasal sampling alone will miss a meaningful subset of carriers. The low MRSA signal is reassuring, yet it calls for continued vigilance, particularly in environments with contact networks that bridge community and healthcare.

The statistically significant discordance between nasal and throat swabs demonstrates that the two anatomical sites are not interchangeable for detecting carriages. Consistently, previous research shows that single-site nasal swabbing underestimates true carriage and that adding throat swabs can increase detection by up to one-third in some populations [17]. Routine reliance on nasal swabs alone risks biased underestimation, particularly in community cohorts where behaviors (e.g., exercise, shared surfaces) may modulate site-specific colonization patterns [17,40]. The biological basis for this discordance likely reflects site-specific ecological niches: the nasal cavity provides a stable, keratinized environment favoring persistent colonization, whereas the throat represents a more dynamic ecosystem influenced by salivary flow, microbiota competition, and environmental exposures [15,18,20,57]. However, the non-negligible proportion of individuals exhibiting throat-only *S. aureus* carriage (n = 6) highlights site-specific colonization dynamics and potential differences in competitive interactions with the oropharyngeal microbiota. This is consistent with previous literature showing that 10–13% of carriers can be throat only [16,17] and that adding throat swabs increases sensitivity by ~26% [16]. Operationally, the incremental workload of adding a throat swab may be justified by improved sensitivity and greater confidence in negative results. Multiple studies therefore recommend combined nasal–throat screening to avoid under-ascertainment of community carriage, thereby addressing the same bias targeted by our paired sampling.

Across sites, *S. aureus* was the most frequent species, with *S. epidermidis* the predominant CNS and additional CNS species detected at lower frequencies. The coexistence of *S. aureus* and CNS in the same samples has important ecological and clinical implications, as CNS may facilitate horizontal gene transfer to more pathogenic species. Nasal samples yielded more isolates overall, mirroring well-described ecological patterns in which the nose harbors higher staphylococcal density and species richness than the throat or skin folds [2,14,21,22]. The higher prevalence in nasal samples likely reflects their adaptation to skin-like environments, whereas the lower throat prevalence may be due to competitive interactions within the oropharyngeal microbiome. CNS (notably *S. epidermidis* and *S. haemolyticus*) often carry mobile genetic elements (e.g., SCCmec) and can act as reservoirs of resistance genes with potential horizontal transfer to *S. aureus* or other pathogens [8,13,21]. In the context of a fitness-center cohort, these findings are biologically plausible. Frequent skin-surface contact, micro-abrasions, and shared equipment create opportunities for microbial exchange; environmental studies of fitness facilities have documented diverse staphylococcal communities on high-touch surfaces [40,41]. *S. aureus*’s desiccation tolerance further supports persistence on fomites between cleaning cycles, potentially seeding transient colonization [6,13]. While the present study was not aimed at linking environmental contamination to individual colonization events, the species distribution and the presence of MDR provide a rationale for enhanced hygiene protocols (e.g., effective surface disinfection, hand hygiene reinforcement, and user education). These findings support the growing recognition of CNS as important components of the staphylococcal ecosystem, highlighting the need for surveillance beyond clinical settings.

In the present study, the susceptibility profile of *S. aureus* isolates was characterized by low levels of antimicrobial resistance: 11 fully susceptible isolates, penicillin G resistance as the predominant phenotype (47%), and rare MDR patterns (6%). This is consistent with the known persistence of penicillinase-mediated resistance in community *S. aureus* and with generally lower resistance burdens in colonizing community strains compared with invasive healthcare-associated isolates [2,12,13,27]. The observed resistance profile (dominant penicillin G resistance with otherwise broad susceptibility and rare MDR) aligns with a 2024 review noting persistent β-lactamase-mediated penicillin resistance in community *S. aureus* but generally favorable susceptibility to non-β-lactams [58]. The PVL-negative MRSA subset also aligns with lower expected resistance multiplicity relative to some epidemic CA-MRSA clones [24,27,59]. The scarcity of PVL and MRSA in our isolates is also consistent with research indicating that, outside outbreak settings, colonizing community strains tend to show a lower virulence gene burden and less extensive resistance than invasive healthcare-associated lineages [60]. Clinically and from a public-health perspective, these data are reassuring, indicating that colonizing strains in this population have not accumulated extensive resistance. However, the presence of even rare MDR isolates highlights the importance of continued surveillance, particularly given the potential for colonizing strains to serve as precursors to infection; moreover, colonization susceptibility may not necessarily mirror resistance in invasive isolates, and selective pressures (e.g., antibiotic exposure, biocide use in gyms, and personal hygiene products) may shift local resistance patterns over time [35,61,62,63,64]. In addition, given the presence of MDR phenotypes among CNS, attention to potential gene flow across staphylococcal species remains warranted [65].

Multivariate regression analyses revealed no significant associations between *S. aureus* carriage (across overall, nasal, or throat outcomes) and age, sex, recent antibiotic therapy, or other clinical/occupational exposures. While point estimates suggested higher odds in men and the highest prevalence in young adults (18–34 years), confidence intervals were wide and overlapped unity, indicating limited precision and a likely sample-size constraint rather than robust null effects. Previous studies have variably reported male sex, younger age, and specific lifestyle factors as correlates of carriage, though findings are heterogeneous and context-dependent [8,12,43,47,66]. Several explanations may account for this discrepancy. First, the relatively small sample size limits statistical power, particularly for detecting moderate associations. Second, the homogeneity of the study population (fitness studio attendees) may reduce variability in key exposures. Third, colonization may be influenced by unmeasured variables such as hygiene behavior, contact networks, or microbiome composition, which were not captured in this study. The borderline sex–age distribution differences at enrollment also caution against over-interpreting subgroup patterns without adequate power. Although no statistically significant associations were identified, descriptive analyses suggested higher carriage prevalence in younger adults (18–34 years) and a potential decline with increasing age, with sex-specific variations in older age groups. Evidence further suggests that host-site biology (e.g., defensins, local milieu) and behavioral factors may overshadow coarse demographics [67,68], which is consistent with our modest AUCs and overall limited discriminative performance with wide confidence intervals. Therefore, these findings should be interpreted with caution, given the modest sample size and corresponding limitations in statistical power and model precision. From a conceptual perspective, these findings support the notion that *S. aureus* colonization in community settings may be only weakly determined by classical demographic or clinical risk factors and instead shaped by complex, context-dependent interactions. This highlights a gap in current epidemiological models and suggests that future research should incorporate behavioral and ecological variables to better explain colonization variability.

In the present study, rep-PCR typing of 52 isolates revealed four similarity clusters (≥85%), with the largest containing 31 isolates and no clear association with anatomical site or demographics. This pattern suggests moderate strain diversity within a shared community environment, compatible with either multiple introductions of common community clones or local circulation with limited clonal dominance. Rep-PCR, while practical for screening, provides low phylogenetic resolution; the absence of site/demographic clustering should therefore be interpreted cautiously [13]. Our rep-PCR finding of moderate strain diversity without clear site/demographic clustering is in line with paired colonization-infection studies showing minimal genetic/phenotypic shifts between carriage and invasive isolates, implicating barrier breach over rapid within-host adaptation [28,69]. The absence of clustering may indicate multiple independent acquisition events rather than localized transmission within the study population. Given modern genomic epidemiology, whole-genome sequencing may better define strain relatedness, detect resistance/virulence genes (e.g., *mecA*, *lukF-PV/lukS-PV*), and assess microtransmission [70]. However, the observed diversity without clear sub-epidemics supports predominantly sporadic progression from colonization to infection, consistent with evidence that *S. aureus* often requires minimal genetic change for invasiveness [28,71].

Importantly, these findings also have methodological implications for community screening strategies: while conventional culture-based approaches remain the standard, molecular diagnostics (e.g., PCR-based assays) could be considered as complementary or alternative tools, particularly for pharyngeal samples where bacterial load may be lower, and culture sensitivity may be reduced [72]. Such methods offer faster turnaround times and potentially higher analytical sensitivity [73], which may improve detection rates of *S. aureus* carriage, including low-density colonization. However, their implementation in community screening programs must be balanced against higher costs, the need for technical infrastructure, and the inability to directly assess phenotypic antimicrobial susceptibility [74]. Future studies should therefore evaluate the cost-effectiveness and diagnostic yield of integrating molecular approaches alongside traditional culture methods in community-based surveillance.

Several limitations should be acknowledged. The cross-sectional design precludes evaluation of temporal dynamics, and the modest sample size limits statistical power, particularly for regression analyses. Additionally, single-site recruitment may constrain generalizability, and self-sampling could introduce variability in specimen quality. Despite these limitations, a key strength is the integration of multi-site sampling, antimicrobial resistance profiling, and molecular typing within a community-based cohort. This comprehensive approach enables a more nuanced characterization of colonization beyond prevalence estimates. The findings offer practical implications: they support multi-site sampling in surveillance, highlight the ecological role of *S. aureus* in resistance dissemination, and point to the need for targeted hygiene interventions in high-contact settings such as fitness facilities. This study adds value by providing species-level and resistance data in a non-clinical population, an area that remains insufficiently characterized.

## 5. Conclusions

In this cross-sectional study of community-dwelling adults in Germany, *S. aureus* carriage was frequent, with the anterior nares as the primary site, but with substantial discordance between nasal and throat colonization. Addressing the study objective, these findings demonstrate that single-site nasal screening underestimates true carriage, as a relevant proportion of individuals would be missed without combined sampling. The low prevalence of MRSA, absence of PVL-positive isolates, and largely favorable antimicrobial susceptibility profiles indicate that colonizing strains in this setting are predominantly non-epidemic and remain broadly susceptible. The coexistence of *S. aureus* with CNS highlights the ecological complexity of colonization and the potential role of commensal species as reservoirs of resistance determinants. The observed moderate genetic diversity without clear clustering by site or demographics suggests heterogeneous acquisition rather than localized transmission. The lack of significant associations with demographic or clinical variables further indicates that colonization in this context is likely shaped by multifactorial and context-dependent influences, although interpretation is limited by modest model performance and statistical power considerations. Collectively, these findings support a shift toward anatomically inclusive and context-sensitive surveillance frameworks while also underscoring that *S. aureus* carriage in community settings is shaped by site-specific ecological dynamics and heterogeneous acquisition rather than simple demographic determinants. This highlights the need for prevention and monitoring strategies that integrate anatomical, ecological, and environmental dimensions while remaining aligned with the constraints of cross-sectional evidence.

## Figures and Tables

**Table 1:** Clinical characteristics and *S. aureus* carriage patterns stratified by sex.

		Overall (n = 100)	Females (n = 65)	Males (n = 35)	*p*-Value
Age (years)		34 (26, 55)	32 (23, 62)	36 (27, 49)	0.9
Age Groups	<18	3 (3.0%)	3 (4.6%)	0 (0%)	0.050
	18–34	50 (50%)	33 (51%)	17 (49%)	
	35–49	16 (16%)	6 (9.2%)	10 (29%)	
	50–64	13 (13%)	8 (12%)	5 (14%)	
	>65	18 (18%)	15 (23%)	3 (8.6%)	
Diabetes I/II		3 (3.0%)	2 (3.1%)	1 (2.9%)	>0.9
Blood pressure medication		14 (14%)	10 (15%)	4 (11%)	0.8
Inpatient treatment		19 (19%)	16 (25%)	3 (8.6%)	0.051
Other chronic condition		0 (0%)	0 (0%)	0 (0%)	>0.9
Antibiotic therapy		15 (15%)	9 (14%)	6 (17%)	0.7
Chronic wounds		2 (2.0%)	1 (1.5%)	1 (2.9%)	>0.9
Acute wounds		6 (6.0%)	2 (3.1%)	4 (11%)	0.2
Catheter therapy		2 (2.0%)	1 (1.5%)	1 (2.9%)	>0.9
Care-home contact (relatives)		8 (8.0%)	6 (9.2%)	2 (5.7%)	0.7
Healthcare worker		27 (27%)	18 (28%)	9 (26%)	0.8
Agriculture contact		4 (4.0%)	2 (3.1%)	2 (5.7%)	0.6
Skin infections		2 (2.0%)	2 (3.1%)	0 (0%)	0.5
Cystic fibrosis		0 (0%)	0 (0%)	0 (0%)	>0.9
Atopic dermatitis		0 (0%)	0 (0%)	0 (0%)	>0.9
Overall *S. aureus* carriage		39 (39%)	24 (37%)	15 (43%)	0.6
Nasal *S. aureus* carriage		33 (33%)	19 (29%)	14 (40%)	0.3
Throat *S. aureus* carriage		19 (19%)	15 (23%)	4 (11%)	0.2
MRSA-positive		3 (3.0%)	3 (4.6%)	0 (0%)	0.5
*S. aureus* carriage pattern	Neither	61 (61%)	41 (63%)	20 (57%)	0.2
	Nasal only	20 (20%)	9 (14%)	11 (31%)	
	Throat only	6 (6.0%)	5 (7.7%)	1 (2.9%)	
	Nasal + Throat	13 (13%)	10 (15%)	3 (8.6%)	

MRSA: methicillin-resistant *Staphylococcus aureus*. Data are presented as median (Q1, Q3) and n (%).

**Table 2:** Resistance profiles of *Staphylococcus aureus* isolates from nasal and throat samples.

Resistance Category	Resistance Profile	Total	Nasal	Throat
Fully susceptible	No resistance detected	11 (35%)	5	6
Single-class resistance	Penicillin G	15 (47%)	10	5
	Tetracycline	1 (3%)	1	0
Two-class resistance	Penicillin G, Erythromycin	1 (3%)	1	0
	Cefotaxime, Erythromycin	1 (3%)	0	1
	Cefotaxime, Penicillin G	1 (3%)	1	0
Multidrug resistance	Penicillin G, Erythromycin, Fusidic acid	1 (3%)	1	0
	Cefotaxime, Tetracycline, Penicillin G, Erythromycin, Fusidic acid	1 (3%)	0	1

Multidrug resistance was defined as resistance to at least one agent in three or more antimicrobial classes. Additional antimicrobial agents tested, for which no resistance was detected among *S. aureus* isolates, included clindamycin, ampicillin–sulbactam, gentamicin, imipenem, ciprofloxacin, moxifloxacin, tigecycline, linezolid, vancomycin, teicoplanin, oxacillin, rifampicin, fosfomycin, and daptomycin.

**Table 3:** Multivariable logistic regression models for key determinants of *S. aureus* carriage across anatomical sites.

	Overall *S. aureus*			Nasal Carriage			Throat Carriage		
	OR	95% CI	*p*-Value	OR	95% CI	*p*-Value	OR	95% CI	*p*-Value
Age (years)	0.98	0.96, 1.01	0.2	0.98	0.95, 1.01	0.2	0.98	0.95, 1.01	0.3
Sex (ref: females)	9.11	0.75, 163	0.10	6.46	0.55, 103	0.2	3.21	0.08, 435	0.6
Recent antibiotic therapy (ref: no)	1.30	0.39, 4.20	0.7	1.30	0.37, 4.25	0.7	0.25	0.01, 1.47	0.2
Age x Sex (ref: females)	0.95	0.88, 1.01	0.12	0.96	0.89, 1.02	0.3	0.94	0.80, 1.04	0.4
Model fit (AIC)	135.8575316			128.2740936			98.3245397		
Model discrimination (AUC)	0.6469105			0.6503844			0.6806368		

CI: confidence interval. OR: odds ratio. Sex was coded with females as the reference category; recent antibiotic therapy was coded with “no” as the reference. The age–sex interaction represents the modification of the age effect among males relative to females. Model diagnostics include model fit (AIC) and model discrimination (AUC), with lower AIC and higher AUC indicating better-performing models.

## Data Availability

The original contributions presented in this study are included in the article/Supplementary Material. Further inquiries can be directed to the corresponding authors.

## Supplementary Materials

The following supporting information can be downloaded at: https://www.mdpi.com/article/10.3390/microorganisms14051053/s1, Figure S1: Rep-PCR analysis. Dendrogram and computer-generated image of rep-PCR band patterns. Part 1: *S. aureus* isolates of the test subjects (No. 1–26); Figure S2: Rep-PCR analysis. Dendrogram and computer-generated image of rep-PCR band patterns. Part 2: *S. aureus* isolates of the test subjects (No. 27–52).

## Abbreviations

The following abbreviations are used in this manuscript:

WHO	World Health Organization
*S. aureus*	*Staphylococcus aureus*
CNS	Coagulase-negative staphylococci
*S. epidermidis*	*Staphylococcus epidermidis*
*S. hominis*	*Staphylococcus hominis*
*S. haemolyticus*	*Staphylococcus haemolyticus*
*S. saprophyticus*	*Staphylococcus saprophyticus*
*S. warneri*	*Staphylococcus warneri*
MRSA	Methicillin-resistant Staphylococcus aureus
CA-MRSA	Community-associated methicillin-resistant Staphylococcus aureus
mecA	Methicillin resistance gene A
PBP2a	Penicillin-binding protein 2a
SCCmec	Staphylococcal cassette chromosome mec
PVL	Panton–Valentine leukocidin
lukF-PV/lukS-PV	Panton–Valentine leukocidin toxin components
REP	Repetitive extragenic palindromic sequences
rep-PCR	Repetitive element polymerase chain reaction
PCR	Polymerase chain reaction
MALDI-TOF MS	Matrix-assisted laser desorption and ionization time-of-flight mass spectrometry
ATCC	American Type Culture Collection
DNA	Deoxyribonucleic acid
MDR	Multidrug-resistant
EUCAST	European Committee on Antimicrobial Susceptibility Testing
CI	Confidence interval
OR	Odds ratio
AIC	Akaike information criterion
AUC	Area under the curve
PABAK	Prevalence- and bias-adjusted kappa
UPGMA	Unweighted pair group method with arithmetic mean
µL	Microliter
