# Supplementary material for: Patterns, Associated Factors, and Anatomical Concordance of Nasal and Throat Staphylococcus aureus Carriage Among Community-Dwelling Adults in Germany
Source: Microorganisms. 2026 May 8;14(5):1053. doi: 10.3390/microorganisms14051053 (PMC13209327; doi:10.3390/microorganisms14051053)
Supplement: Supplementary file 1 [file microorganisms-14-01053-s001.zip › Supplementary Materials_Martens et al _1_microorganisms2026.pdf]

Supplementary Materials (Figure S1 and Figure S2)

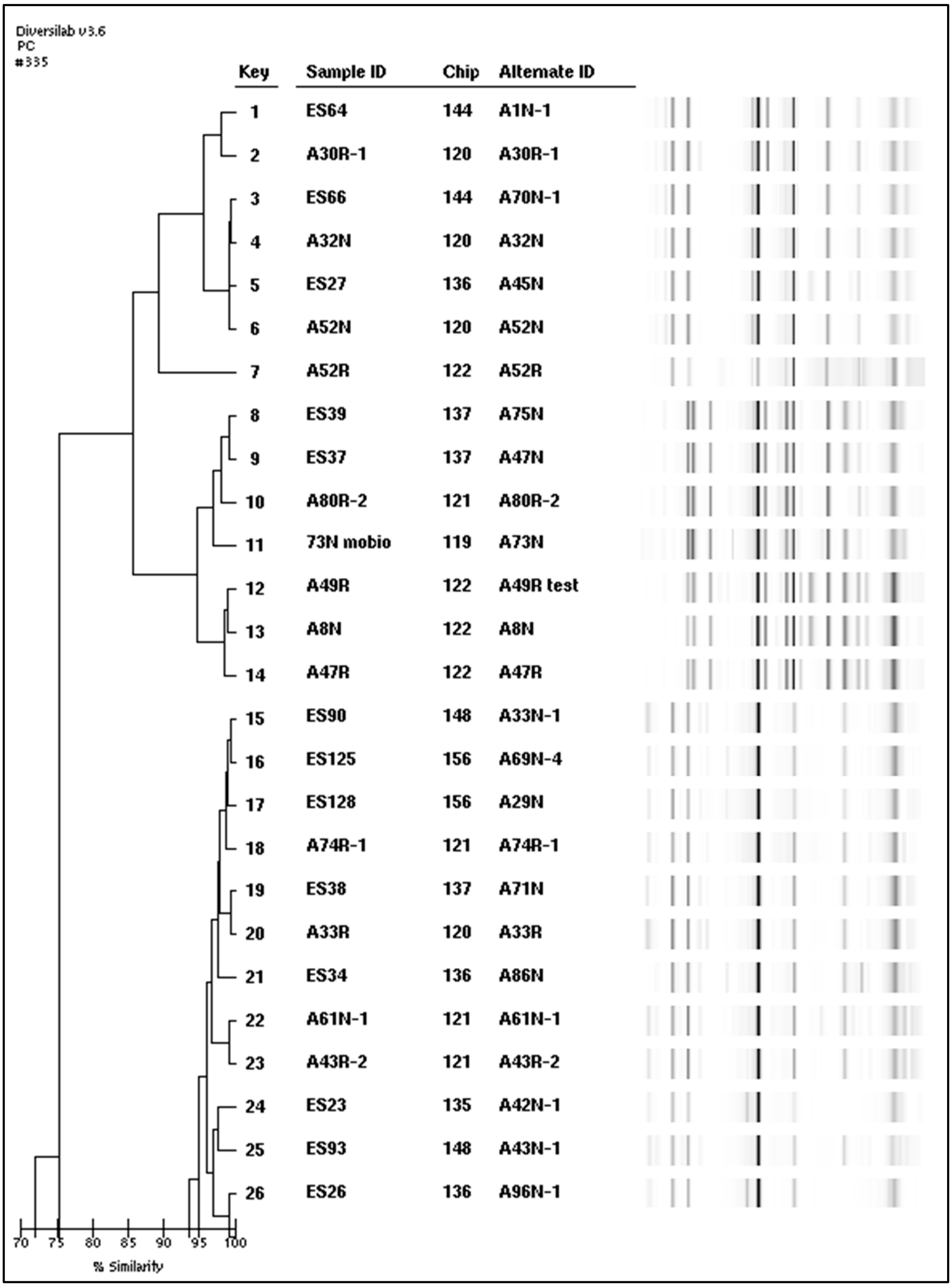

Figure S1. Rep-PCR analysis. Dendrogram and computer-generated image of rep-PCR band patterns. Part 1: *S. aureus* isolates of the test subjects (No. 1-26)

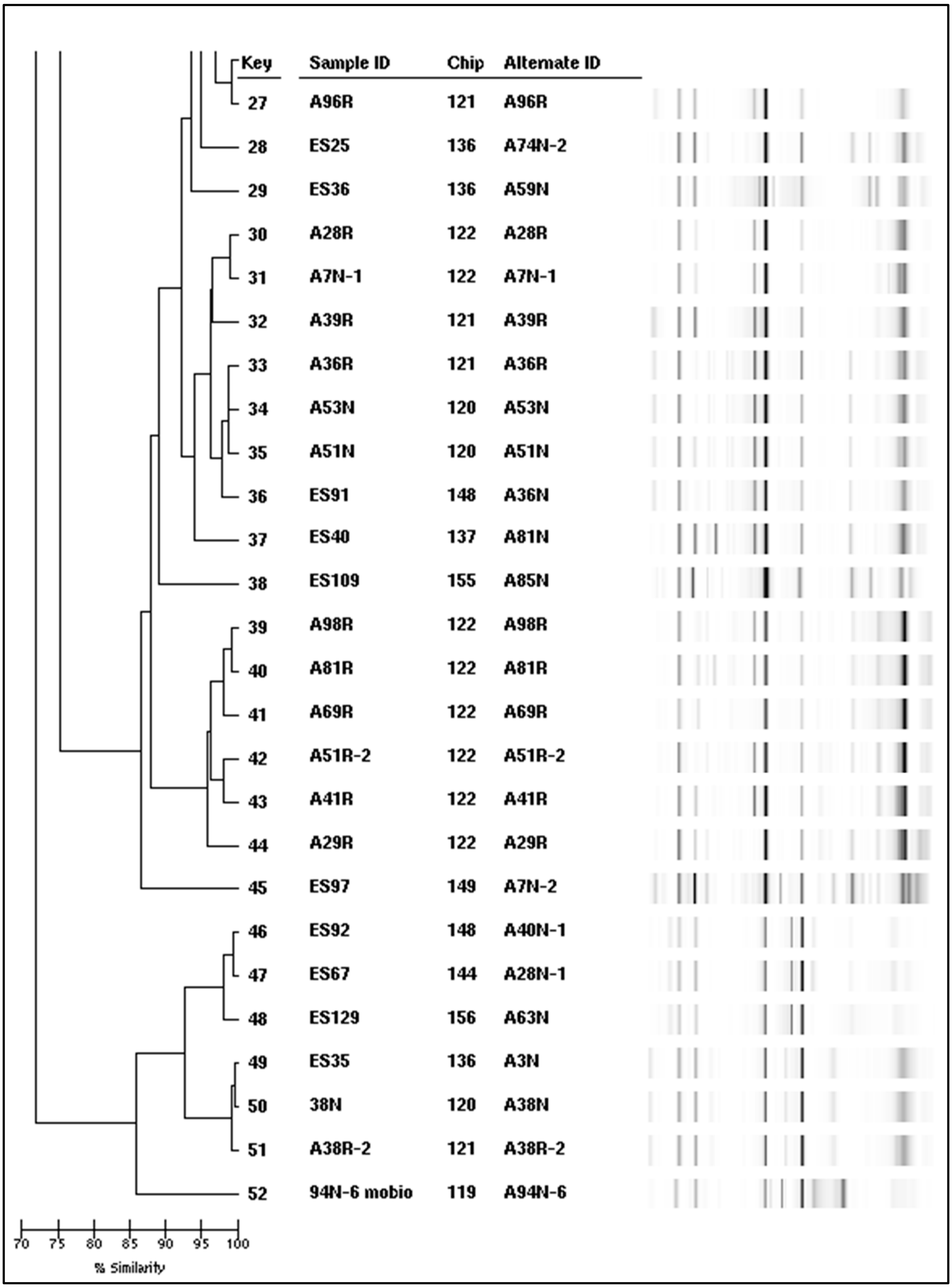

Figure S2. Rep-PCR analysis. Dendrogram and computer-generated image of rep-PCR band patterns. Part 2: *S. aureus* isolates of the test subjects (No. 27-52)
